# Crystal structure of *trans*-(1,8-dibutyl-1,3,6,8,10,13-hexa­aza­cyclo­tetra­decane-κ^4^*N*^3^,*N*^6^,*N*^10^,*N*^13^)bis­(perchlorato-κ*O*)nickel(II) from synchrotron data

**DOI:** 10.1107/S2056989026001817

**Published:** 2026-02-24

**Authors:** Dongwon Kim, Dae-Woong Kim, Dohyun Moon

**Affiliations:** a Beamline Department, Pohang Acceleratory Laboratory, Pohang 37673, Republic of Korea; Universidad de la Repüblica, Uruguay

**Keywords:** crystal structure, aza­macrocyclic ligand, Jahn–Teller distortion, hydrogen bonds, synchrotron data

## Abstract

The crystal structure of the nickel(II) macrocyclic title complex, *trans*- [Ni(ClO_4_)_2_(C_16_H_38_N_6_)], was analyzed using synchrotron radiation. The coordination environment consists of four nitro­gen atoms from the ligand [Ni—N = 1.9382 (16), 1.9378 (17) Å] and two perchlorate oxygen atoms [Ni—O = 2.878 (3) Å], adopting an octa­hedral geometry with slight tetra­gonal distortion. The structural comparison with its Cu^II^ analogue reveals a notable elongation in the axial Ni—O bonds, which is attributed to the steric hindrance of the macrocyclic ligand and weak axial coordination. Supra­molecular inter­actions involving hydrogen bonding further consolidate the three-dimensional crystal packing.

## Abstract 2

The nickel(II) macrocyclic title complex shows centrosymmetric octa­hedral coordination with elongated axial Ni—O bonds. Unlike the Cu^II^ analogue, it forms a uniform hydrogen-bonded three-dimensional supra­molecular network, as revealed by synchrotron crystallography.

## 1. Chemical context

Macrocyclic transition-metal complexes are of great inter­est due to their versatile applications in catalysis, mol­ecular recognition, and supra­molecular assembly (Wang, 2024 ▸). The coordination environment of these complexes is strongly influenced by the metal center, affecting their structural and electronic properties (He *et al.*, 2012 ▸). Previously, the Cu^II^ analogue of the title complex was reported, exhibiting Jahn–Teller distortion, which resulted in an asymmetric elongation of the axial Cu—O bonds (Kim *et al.*, 2015 ▸). By contrast, Ni^II^, with its *d*^8^ electronic configuration, does not undergo Jahn–Teller distortion, generally leading to a more symmetric octa­hedral geometry (Chandrasekhar *et al.*, 2016 ▸). In the present work, the ligand 1,8-dibutyl-1,3,6,8,10,13-hexa­aza­cyclo­tetra­decane was specifically selected to investigate the structural influence of the bulky *N*-butyl substituents. This design allows for an examination of how steric hindrance, distinct from the electronic Jahn–Teller effect observed in the Cu^II^ analogue, modulates the axial coordination environment. Consequently, we report the crystal structure of the Ni^II^ analogue, focusing on how metal substitution and steric factors collectively influence the coordination geometry and supra­molecular inter­actions. The structure is consolidated by hydrogen bonding, forming a three-dimensional network (Table 1 ▸), providing insights into the structural role of substituent effects in macrocyclic complexes.
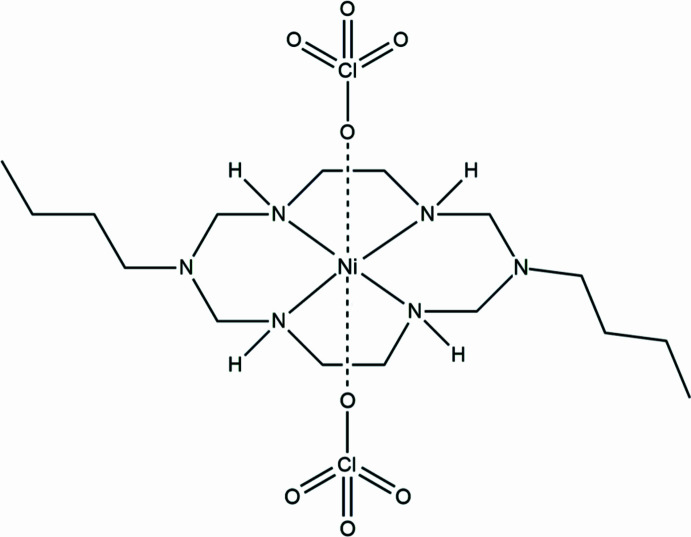


## 2. Structural commentary

**Figure 1:**
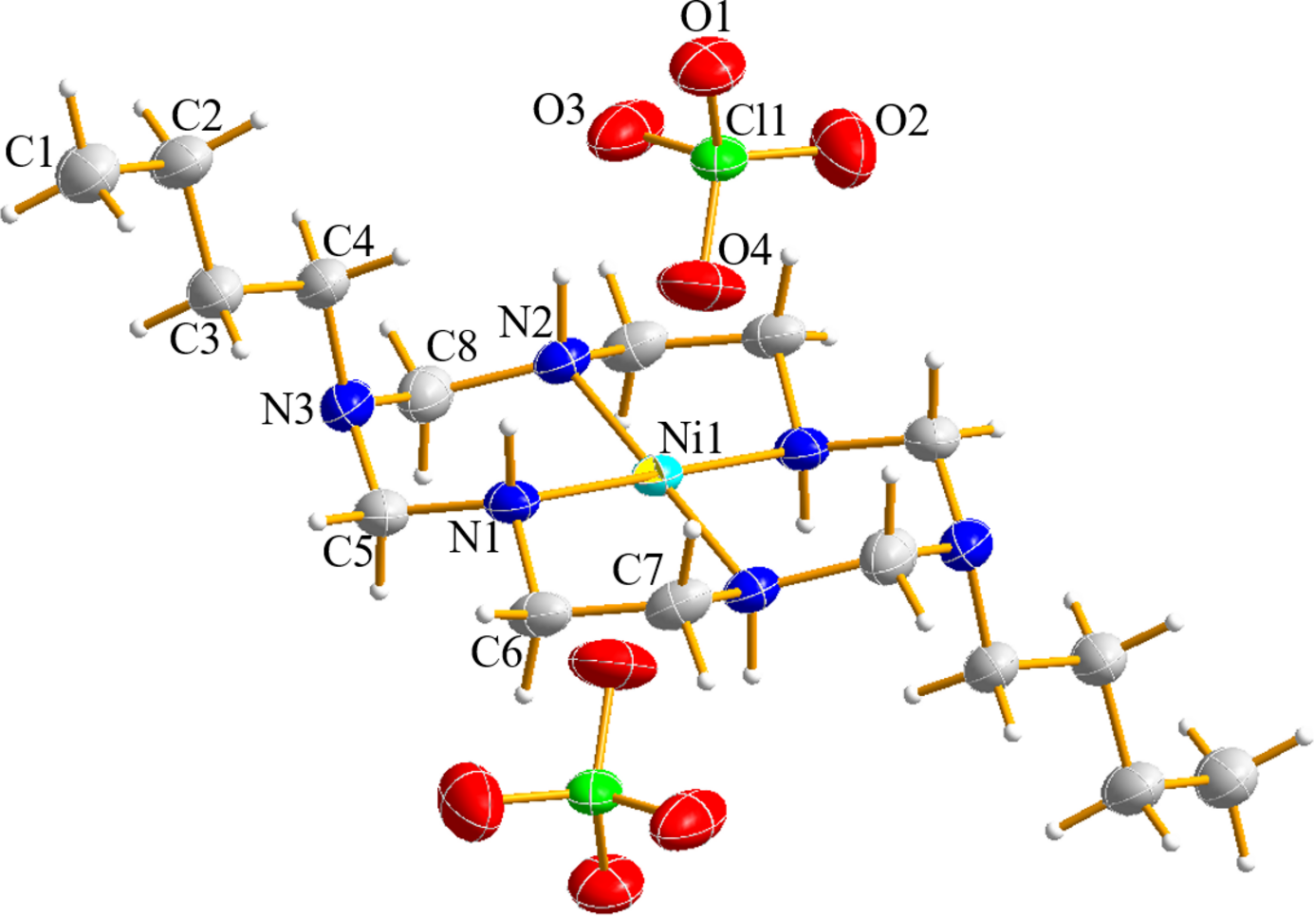
The asymmetric unit of (I) expanded to show the complete nickel(II) ion coordination sphere with displacement ellipsoids drawn at the 30% probability level.

The Ni^II^ center in the title complex adopts an octa­hedral coordination geometry, with four nitro­gen donors in the equatorial plane and two perchlorate oxygen atoms in the axial positions. The Ni—N bond lengths [1.9382 (16), 1.9378 (17) Å] are shorter than those in the Cu^II^ analogue [2.010 (4) Å], whereas the Ni—O bond [2.878 (3) Å] is longer than the Cu—O bond [2.569 (1) Å]. This elongated distance suggests a weak axial inter­action, likely electrostatic in nature, rather than a strong covalent coordination bond. Unlike the Cu^II^ analogue governed by the Jahn–Teller effect, the long Ni—O distance in the title complex is primarily a consequence of the steric requirements of the *N*-butyl substituents, which limit the approach of the weakly coordinating perchlorate anions. The coordination angles reflect a slightly distorted octa­hedral environment, with N—Ni—N angles close to 90° and O—Ni—N angles of 78.12 (8) and 94.60 (8)° (Fig. 1 ▸). Compared to the Cu^II^ complex, which exhibits N—Cu—N angles of 87.68 (8) and 92.32 (8)°, the Ni^II^ structure remains more symmetrical (Kim *et al.*, 2015 ▸). These structural features are further reflected in the supra­molecular packing, particularly in the hydrogen-bonding inter­actions described below.

## 3. Supra­molecular features

**Figure 2:**
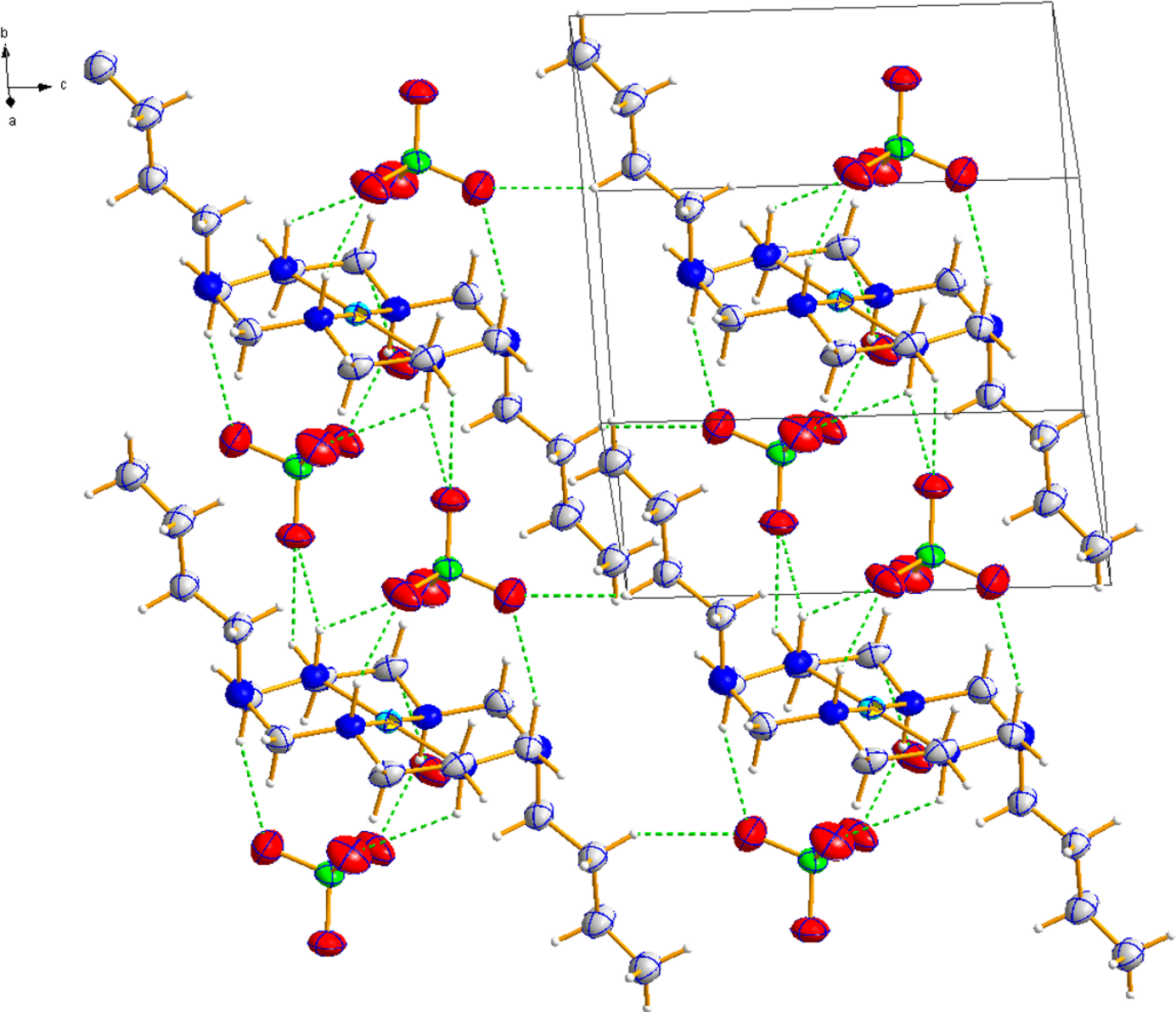
The crystal packing in title compound. Dashed lines represent N—H⋯O and C—H⋯O inter­actions.

The crystal packing of the title complex is primarily governed by hydrogen bonding inter­actions, which contribute to the formation of a three-dimensional supra­molecular network (Fig. 2 ▸). The perchlorate anions play a key role in consolidating the structure by accepting hydrogen bonds from both the ligand and alkyl groups. A notable N—H⋯O inter­action (H⋯O = 2.08 Å, ∠DHA = 156.8°) is observed, in addition to several C—H⋯O contacts, as summarized in Table 1 ▸. Unlike the previously reported Cu^II^ complex, where significant Jahn–Teller distortion resulted in asymmetric hydrogen-bonding patterns, the Ni^II^ complex exhibits a more uniform hydrogen-bonding network compared to the Cu^II^ analogue. This leads to a denser and more compact mol­ecular arrangement, contributing to the structural cohesiveness of the crystal packing. These findings highlight how metal substitution influences supra­molecular assembly, affecting hydrogen-bonding patterns and crystal packing efficiency.

## 4. Database survey

A search of the Cambridge Structural Database (CSD, version 6.00 with updates through April 2025; Groom *et al.*, 2016 ▸) was conducted using ConQuest, focusing on metal complexes of macrocyclic ligands structurally related to cyclam. Among 160 identified complexes (93 Ni, 66 Cu, and 1 Au), no exact structural match to the title nickel(II) complex was found, confirming its novelty. Furthermore, an analysis of the structural parameters within the identified Ni^II^ subset reveals that axial Ni—O distances vary significantly depending on the steric crowding of the ligand. In particular, complexes with bulky substituents often exhibit elongated axial inter­actions exceeding 2.6 Å, similar to the value observed in the title compound [2.878 (3) Å]. This supports the attribution of the long Ni—O distance to steric hindrance rather than inherent electronic effects.

## 5. Synthesis and crystallization

The title nickel(II) complex was prepared as follows. Ethyl­enedi­amine (3.4 mL, 0.05 mol), paraformaldehyde (3.0 g, 0.10 mol), and butyl­amine (3.7 g, 0.05 mol) were slowly added to a stirred solution of NiCl_2_·6H_2_O (5.95 g, 0.025 mol) in methanol (50 mL). The mixture was heated to reflux for 1 day under a nitro­gen atmosphere. After cooling to room temperature, perchloric acid (HClO_4_, 70%, 15 mL) was added dropwise to the reaction mixture with stirring. A pale-yellow precipitate formed immediately, which was collected by filtration and sequentially washed with H_2_O, methanol, and diethyl ether. The resulting solid was then redissolved in aceto­nitrile, and deionized water was carefully layered over the solution. Slow diffusion of water into the aceto­nitrile layer over several days afforded yellow block-shaped crystals suitable for X-ray diffraction. Yield: 9.91 g (70%). **Safety note**: Although we have experienced no problem with the compounds reported in this study, perchlorate salts of metal complexes are often explosive and should be handled with great caution.

## 6. Refinement

Crystal data, data collection and structure refinement details are summarized in Table 2 ▸. To maximize data completeness, datasets from two separate measurements were merged, resulting in a completeness of 98.6%. The remaining missing reflections are attributed to the geometric constraints of the single-axis goniometer at the synchrotron beamline, which limits full coverage of the reciprocal space. All H atoms were placed in geometrically idealized positions and constrained to ride on their parent atoms, with C—H distances of 0.97–0.98 Å and an N—H distance of 0.99 Å with *U*_iso_(H) values of 1.2 or 1.5 *U*_eq_ of the parent atoms.

## Supplementary Material

Crystal structure: contains datablock(s) I. DOI: 10.1107/S2056989026001817/ny2019sup1.cif

Structure factors: contains datablock(s) I. DOI: 10.1107/S2056989026001817/ny2019Isup2.hkl

CCDC reference: 2531895

Additional supporting information:  crystallographic information; 3D view; checkCIF report

## Figures and Tables

**Table 1:** Hydrogen-bond geometry (Å, °)

*D*—H⋯*A*	*D*—H	H⋯*A*	*D*⋯*A*	*D*—H⋯*A*
N1—H1⋯O1^i^	0.99	2.24	3.008 (2)	134
N1—H1⋯O4^ii^	0.99	2.50	3.119 (3)	120
N2—H2⋯O3^ii^	0.99	2.08	3.014 (2)	157
C3—H3*B*⋯O2^iii^	0.98	2.63	3.439 (3)	140
C5—H5*A*⋯O2	0.98	2.59	3.506 (3)	155
C6—H6*B*⋯O1^i^	0.98	2.51	3.072 (2)	116
C7—H7*B*⋯O3^iv^	0.98	2.47	3.220 (3)	133

Symmetry codes: (i) 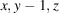; (ii) 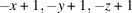; (iii) 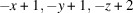; (iv) 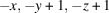.

**Table 2:** Experimental details

Crystal data	
Chemical formula	[Ni(ClO_4_)_2_(C_16_H_38_N_6_)]
*M*_r_	572.13
Crystal system, space group	Triclinic, *P* 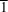
Temperature (K)	220
*a*, *b*, *c* (Å)	8.2510 (16), 8.4230 (17), 10.097 (2)
α, β, γ (°)	92.57 (3), 95.31 (3), 117.49 (3)
*V* (Å^3^)	616.8 (3)
*Z*	1
Radiation type	Synchrotron, λ = 0.700 Å
μ (mm^−1^)	1.01
Crystal size (mm)	0.08 × 0.06 × 0.01

Data collection	
Diffractometer	Rayonix MX225HS CCD area detector
Absorption correction	Empirical (using intensity measurements) (*HKL3000sm *SCALEPACK**; Otwinowski *et al.*, 2003 ▸)
*T*_min_, *T*_max_	0.936, 1.000
No. of measured, independent and observed [*I* > 2σ(*I*)] reflections	6999, 3509, 3287
*R*_int_	0.026
(sin θ/λ)_max_ (Å^−1^)	0.704

Refinement	
*R*[*F*^2^ > 2σ(*F*^2^)], *wR*(*F*^2^), *S*	0.058, 0.147, 1.18
No. of reflections	3509
No. of parameters	152
H-atom treatment	H-atom parameters constrained
Δρ_max_, Δρ_min_ (e Å^−3^)	0.47, −1.81

Computer programs: *PAL BL2D-SMDC Program* (Shin *et al.*, 2025 ▸), *HKL3000sm* (Otwinowski *et al.*, 2003 ▸), *SHELXT2018* (Sheldrick, 2015*a* ▸), *SHELXL2018* (Sheldrick, 2015*b* ▸), *DIAMOND 4* (Putz & Brandenburg, 2014 ▸) and *publCIF* (Westrip, 2010 ▸).

## supplementary crystallographic information

### trans-(1,8-Dibutyl-1,3,6,8,10,13-hexaazacyclotetradecane-κ4N3,N6,N10,N13)bis(perchlorato-κO)nickel(II) Crystal data

**Table:**

[Ni(ClO_4_)_2_(C_16_H_38_N_6_)]	*Z* = 1
*M**_r_* = 572.13	*F*(000) = 302
Triclinic, *P*1	*D*_x_ = 1.540 Mg m^−^^3^
*a* = 8.2510 (16) Å	Synchrotron radiation, λ = 0.700 Å
*b* = 8.4230 (17) Å	Cell parameters from 12822 reflections
*c* = 10.097 (2) Å	θ = 0.4–29.5°
α = 92.57 (3)°	µ = 1.01 mm^−^^1^
β = 95.31 (3)°	*T* = 220 K
γ = 117.49 (3)°	Plate, dark yellow
*V* = 616.8 (3) Å^3^	0.08 × 0.06 × 0.01 mm

### trans-(1,8-Dibutyl-1,3,6,8,10,13-hexaazacyclotetradecane-κ4N3,N6,N10,N13)bis(perchlorato-κO)nickel(II) Data collection

**Table:**

Rayonix MX225HS CCD area detector diffractometer	3287 reflections with *I* > 2σ(*I*)
Radiation source: PLSII 2D bending magnet	*R*_int_ = 0.026
ω scan	θ_max_ = 29.5°, θ_min_ = 2.0°
Absorption correction: empirical (using intensity measurements) (*HKL3000sm Scalepack*; Otwinowski *et al.*, 2003)	*h* = −11→11
*T*_min_ = 0.936, *T*_max_ = 1.000	*k* = −11→11
6999 measured reflections	*l* = −14→14
3509 independent reflections	

### trans-(1,8-Dibutyl-1,3,6,8,10,13-hexaazacyclotetradecane-κ4N3,N6,N10,N13)bis(perchlorato-κO)nickel(II) Refinement

**Table:**

Refinement on *F*^2^	0 restraints
Least-squares matrix: full	Hydrogen site location: inferred from neighbouring sites
*R*[*F*^2^ > 2σ(*F*^2^)] = 0.058	H-atom parameters constrained
*wR*(*F*^2^) = 0.147	*w* = 1/[σ^2^(*F*_o_^2^) + (0.0999*P*)^2^ + 0.0934*P*] where *P* = (*F*_o_^2^ + 2*F*_c_^2^)/3
*S* = 1.18	(Δ/σ)_max_ < 0.001
3509 reflections	Δρ_max_ = 0.47 e Å^−^^3^
152 parameters	Δρ_min_ = −1.80 e Å^−^^3^

### trans-(1,8-Dibutyl-1,3,6,8,10,13-hexaazacyclotetradecane-κ4N3,N6,N10,N13)bis(perchlorato-κO)nickel(II) Special details

**Table:**

Geometry. All esds (except the esd in the dihedral angle between two l.s. planes) are estimated using the full covariance matrix. The cell esds are taken into account individually in the estimation of esds in distances, angles and torsion angles; correlations between esds in cell parameters are only used when they are defined by crystal symmetry. An approximate (isotropic) treatment of cell esds is used for estimating esds involving l.s. planes.

### trans-(1,8-Dibutyl-1,3,6,8,10,13-hexaazacyclotetradecane-κ4N3,N6,N10,N13)bis(perchlorato-κO)nickel(II) Fractional atomic coordinates and isotropic or equivalent isotropic displacement parameters (Å^2^)

**Table:**

	*x*	*y*	*z*	*U*_iso_*/*U*_eq_	
Ni1	0.500000	0.500000	0.500000	0.02865 (13)	
N1	0.4287 (2)	0.3527 (2)	0.64745 (15)	0.0329 (3)	
H1	0.456229	0.251823	0.628789	0.049*	
N2	0.7582 (2)	0.6199 (2)	0.57271 (16)	0.0333 (3)	
H2	0.808852	0.537674	0.549442	0.040*	
C1	0.7678 (4)	−0.0061 (4)	0.9829 (3)	0.0519 (5)	
H1A	0.835113	−0.074717	0.979837	0.078*	
H1B	0.638215	−0.084755	0.953240	0.078*	
H1C	0.782480	0.045415	1.073839	0.078*	
C2	0.8423 (3)	0.1436 (4)	0.8919 (2)	0.0498 (5)	
H2A	0.827911	0.090796	0.800375	0.060*	
H2B	0.974238	0.220040	0.920404	0.060*	
C3	0.7450 (3)	0.2592 (3)	0.8930 (2)	0.0449 (4)	
H3A	0.611501	0.180816	0.878160	0.054*	
H3B	0.775161	0.326538	0.981259	0.054*	
C4	0.7984 (3)	0.3901 (3)	0.7875 (2)	0.0418 (4)	
H4A	0.933003	0.458707	0.796080	0.050*	
H4B	0.755647	0.322150	0.698952	0.050*	
C5	0.5247 (3)	0.4391 (3)	0.78487 (19)	0.0405 (4)	
H5A	0.491830	0.533258	0.809882	0.049*	
H5B	0.479571	0.348424	0.848515	0.049*	
C6	0.2252 (3)	0.2719 (3)	0.6434 (2)	0.0386 (4)	
H6A	0.191040	0.359483	0.681686	0.046*	
H6B	0.178746	0.166550	0.694201	0.046*	
C7	0.1469 (2)	0.2181 (3)	0.4983 (2)	0.0397 (4)	
H7A	0.168898	0.120508	0.462803	0.048*	
H7B	0.013827	0.176938	0.487386	0.048*	
C8	0.8030 (3)	0.6671 (3)	0.7210 (2)	0.0415 (4)	
H8A	0.937104	0.725037	0.744424	0.050*	
H8B	0.761373	0.754607	0.746459	0.050*	
Cl1	0.33108 (7)	0.79716 (6)	0.65106 (4)	0.03772 (14)	
O1	0.3222 (3)	0.9624 (2)	0.6678 (2)	0.0539 (4)	
O2	0.3190 (4)	0.7220 (3)	0.7760 (2)	0.0729 (6)	
O3	0.1753 (3)	0.6698 (3)	0.5582 (2)	0.0663 (6)	
N3	0.7217 (2)	0.5168 (3)	0.79660 (16)	0.0404 (4)	
O4	0.4977 (3)	0.8233 (3)	0.6028 (2)	0.0689 (6)	

### trans-(1,8-Dibutyl-1,3,6,8,10,13-hexaazacyclotetradecane-κ4N3,N6,N10,N13)bis(perchlorato-κO)nickel(II) Atomic displacement parameters (Å^2^)

**Table:**

	*U*^11^	*U*^22^	*U*^33^	*U*^12^	*U*^13^	*U*^23^
Ni1	0.02951 (18)	0.03165 (19)	0.03204 (18)	0.02014 (14)	0.00520 (11)	0.00361 (11)
N1	0.0361 (7)	0.0376 (7)	0.0368 (7)	0.0259 (6)	0.0100 (5)	0.0083 (5)
N2	0.0313 (6)	0.0351 (7)	0.0401 (7)	0.0214 (6)	0.0027 (5)	0.0034 (5)
C1	0.0587 (13)	0.0598 (13)	0.0517 (12)	0.0386 (12)	0.0099 (10)	0.0134 (10)
C2	0.0527 (11)	0.0656 (14)	0.0514 (11)	0.0427 (11)	0.0119 (9)	0.0172 (10)
C3	0.0540 (11)	0.0587 (12)	0.0385 (9)	0.0393 (10)	0.0094 (8)	0.0102 (8)
C4	0.0455 (10)	0.0563 (11)	0.0383 (9)	0.0357 (9)	0.0063 (7)	0.0091 (8)
C5	0.0459 (10)	0.0550 (11)	0.0360 (8)	0.0359 (9)	0.0084 (7)	0.0058 (7)
C6	0.0347 (8)	0.0420 (9)	0.0508 (10)	0.0251 (8)	0.0157 (7)	0.0158 (7)
C7	0.0296 (7)	0.0365 (9)	0.0567 (11)	0.0182 (7)	0.0052 (7)	0.0096 (7)
C8	0.0450 (9)	0.0430 (10)	0.0407 (9)	0.0260 (8)	−0.0023 (7)	−0.0027 (7)
Cl1	0.0447 (3)	0.0446 (3)	0.0378 (2)	0.0332 (2)	0.00443 (17)	−0.00001 (17)
O1	0.0660 (10)	0.0446 (8)	0.0669 (10)	0.0390 (8)	0.0103 (8)	0.0034 (7)
O2	0.1124 (19)	0.0827 (15)	0.0512 (10)	0.0662 (15)	0.0185 (11)	0.0200 (10)
O3	0.0649 (11)	0.0736 (12)	0.0720 (12)	0.0503 (10)	−0.0193 (9)	−0.0280 (10)
N3	0.0454 (8)	0.0505 (9)	0.0358 (7)	0.0323 (8)	0.0012 (6)	0.0022 (6)
O4	0.0544 (10)	0.0839 (14)	0.0850 (14)	0.0444 (10)	0.0237 (10)	0.0001 (11)

### trans-(1,8-Dibutyl-1,3,6,8,10,13-hexaazacyclotetradecane-κ4N3,N6,N10,N13)bis(perchlorato-κO)nickel(II) Geometric parameters (Å, º)

**Table:**

Ni1—N2^i^	1.9378 (17)	C3—C4	1.514 (3)
Ni1—N2	1.9378 (17)	C4—N3	1.474 (3)
Ni1—N1^i^	1.9381 (16)	C5—N3	1.435 (3)
Ni1—N1	1.9382 (16)	C6—C7	1.505 (3)
N1—C6	1.488 (2)	C8—N3	1.426 (3)
N1—C5	1.502 (3)	Cl1—O4	1.4252 (18)
N2—C7^i^	1.481 (3)	Cl1—O2	1.428 (2)
N2—C8	1.499 (3)	Cl1—O1	1.4309 (16)
C1—C2	1.519 (3)	Cl1—O3	1.448 (2)
C2—C3	1.522 (3)		
			
N2^i^—Ni1—N2	180.00 (9)	N3—C4—C3	113.37 (17)
N2^i^—Ni1—N1^i^	93.03 (7)	N3—C5—N1	114.13 (15)
N2—Ni1—N1^i^	86.97 (7)	N1—C6—C7	106.18 (15)
N2^i^—Ni1—N1	86.97 (7)	N2^i^—C7—C6	106.55 (16)
N2—Ni1—N1	93.03 (7)	N3—C8—N2	113.97 (16)
N1^i^—Ni1—N1	180.0	O4—Cl1—O2	109.21 (15)
C6—N1—C5	111.18 (15)	O4—Cl1—O1	111.56 (13)
C6—N1—Ni1	108.11 (12)	O2—Cl1—O1	109.53 (13)
C5—N1—Ni1	117.48 (13)	O4—Cl1—O3	109.48 (14)
C7^i^—N2—C8	110.58 (16)	O2—Cl1—O3	107.61 (17)
C7^i^—N2—Ni1	108.04 (11)	O1—Cl1—O3	109.36 (12)
C8—N2—Ni1	117.14 (13)	C8—N3—C5	113.03 (16)
C1—C2—C3	112.53 (19)	C8—N3—C4	114.63 (17)
C4—C3—C2	112.59 (18)	C5—N3—C4	116.49 (18)

Symmetry code: (i) −*x*+1, −*y*+1, −*z*+1.

### trans-(1,8-Dibutyl-1,3,6,8,10,13-hexaazacyclotetradecane-κ4N3,N6,N10,N13)bis(perchlorato-κO)nickel(II) Hydrogen-bond geometry (Å, º)

**Table:**

*D*—H···*A*	*D*—H	H···*A*	*D*···*A*	*D*—H···*A*
N1—H1···O1^ii^	0.99	2.24	3.008 (2)	134
N1—H1···O4^i^	0.99	2.50	3.119 (3)	120
N2—H2···O3^i^	0.99	2.08	3.014 (2)	157
C3—H3*B*···O2^iii^	0.98	2.63	3.439 (3)	140
C5—H5*A*···O2	0.98	2.59	3.506 (3)	155
C6—H6*B*···O1^ii^	0.98	2.51	3.072 (2)	116
C7—H7*B*···O3^iv^	0.98	2.47	3.220 (3)	133

Symmetry codes: (i) −*x*+1, −*y*+1, −*z*+1; (ii) *x*, *y*−1, *z*; (iii) −*x*+1, −*y*+1, −*z*+2; (iv) −*x*, −*y*+1, −*z*+1.
